# Host-Directed Antivirals: A Realistic Alternative to Fight Zika Virus

**DOI:** 10.3390/v10090453

**Published:** 2018-08-24

**Authors:** Juan-Carlos Saiz, Nereida Jiménez de Oya, Ana-Belén Blázquez, Estela Escribano-Romero, Miguel A. Martín-Acebes

**Affiliations:** Department of Biotechnology, Instituto Nacional de Investigación y Tecnología Agraria y Alimentaria (INIA), 28040 Madrid, Spain; jdeoya@inia.es (N.J.d.O.); blazquez@inia.es (A.-B.B.); eescribano@inia.es (E.E.-R.)

**Keywords:** flavivirus, Zika virus, therapy, host-directed antivirals

## Abstract

Zika virus (ZIKV), a mosquito-borne flavivirus, was an almost neglected pathogen until its introduction in the Americas in 2015, where it has been responsible for a threat to global health, causing a great social and sanitary alarm due to its increased virulence, rapid spread, and an association with severe neurological and ophthalmological complications. Currently, no specific antiviral therapy against ZIKV is available, and treatments are palliative and mainly directed toward the relief of symptoms, such as fever and rash, by administering antipyretics, anti-histamines, and fluids for dehydration. Nevertheless, lately, search for antivirals has been a major aim in ZIKV investigations. To do so, screening of libraries from different sources, testing of natural compounds, and repurposing of drugs with known antiviral activity have allowed the identification of several antiviral candidates directed to both viral (structural proteins and enzymes) and cellular elements. Here, we present an updated review of current knowledge about anti-ZIKV strategies, focusing on host-directed antivirals as a realistic alternative to combat ZIKV infection.

## 1. Introduction

Since the beginning of the 21st century, a number of infectious disease threats have emerged that demand a global response. Among them, severe acute respiratory syndrome virus, avian influenza in humans, pandemic influenza A (H1N1), Middle East respiratory syndrome coronavirus, chikungunya virus, and Ebola virus have been the most threatening ones. Nonetheless, the emergency of a vector-borne virus, Zika virus (ZIKV), which is responsible for congenital malformations and other neurological and ophthalmological disorders, was hard to predict.

ZIKV is a mosquito-borne virus belonging to the Spondweni serocomplex in the genus *Flavivirus* of the family *Flaviviridae* [1]. The virus has been isolated from various mosquito species, although it seems that the natural transmission vectors are mosquitoes of the genus *Aedes* [2,3]. Besides mosquito bites, viral direct human-to-human transmission can occur perinatally, sexually, and through breastfeeding and blood transfusion [4]. The ZIKV genome is a single-stranded RNA molecule (≈10.7 kb) of positive polarity encoding a single open reading frame (ORF) flanked by two untranslated regions at the 5′ and 3′ ends [5].

ZIKV was first isolated from the serum of a monkey in 1947, and one year later from *Aedes africanus* mosquitoes caught in the same area, the Zika forest [6]. Until it was detected in Asia in the 1980s, the virus had been confined to Africa. Later on, human outbreaks were reported in the Pacific islands, Micronesia in 2007 and, then, in French Polynesia in 2013 [4]. The natural course of ZIKV infection was usually asymptomatic or produce a relatively mild illness and an uneventful recovery [7], hence, the virus was considered an almost neglected pathogen until its recent introduction into the Americas in 2015, when it became a threat to global health, showing increased virulence, rapid spread, and an association with severe neurological complications such as an unexpected rise of microcephaly cases in fetuses and newborns and a remarkable increase in Guillain-Barré syndrome (GBS) cases [8]. This drove the World Health Organization (WHO) to declare a public health emergency of international concern (PHEIC) in 2016 [9].

ZIKV is a neurotropic virus with a wide tissue tropism [10,11,12], including reproductive tissues and organs. In males, ZIKV can infect testes, the prostate, and seminal vesicles [12,13], and in females it can infect the vagina, uterus, vaginal epithelium, uterine fibroblasts, Hofbauer cells, trophoblasts, and endothelial cells from the placenta [12,14]. ZIKV has also been detected in the cornea, neurosensory retina, optic nerve, aqueous humor, and tears [15]. Because of this, ZIKV infection can lead to severe neurological and ophthalmological disorders.

Lack of effective prophylactics, vaccines, or therapeutics hampers the fight against ZIKV. Consequently, inactivated viruses, live vector-based, and nucleic acid (DNA or RNA)-based candidates, subunit elements, virus-like particles, and recombinant viruses are been rehearsed, some of them already in clinical trials [16]. Likewise, many different compounds are being tested as possible therapeutic agents against ZIKV that target either viral or cellular components.

The present review discusses recent advances in the design and development of antivirals and therapeutics for ZIKV infection, focusing in those directed against host factors needed for the viral life cycle as a realistic alternative for the treatment of ZIKV infection.

## 2. Therapeutic Approaches

Since the recent outbreak in 2015 in the Americas, a quite high number of possible antiviral candidates are being tested *in vitro* and *in vivo*. However, until now, no specific therapy has been approved against any flavivirus [17], including ZIKV [18], and, thus, current treatments are mainly directed toward the relief of symptoms, such as fever and rash, by administering antipyretics, anti-histamines, and fluids for dehydration [15]. Nevertheless, it should be noted that some commonly used drugs, such as acetylsalicylic acid, are contraindicated in ZIKV-infected patients, since they increase the risk of internal bleeding, and other arboviruses (dengue or chikungunya viruses) that can co-infect the patients may produce hemorrhages [3].

Due to the natural course of ZIKV infection, which is usually asymptomatic or produce a relatively mild illness and an uneventful recovery, when facing anti-ZIKV strategies, a very important point to take into account is the main target population that would benefit from it, namely immunocompromised patients and pregnant women and their fetuses [4]. In this sense, only for some of the tested drugs their safety profiles are known [19]. However, in cases of Food and Drug Administration (FDA) (https://www.drugs.com/) category B compounds (*animal reproduction studies have failed to demonstrate a risk to the fetus and there are no adequate and well-controlled studies in pregnant women*), or even in those of category C (*animal reproduction studies have shown an adverse effect on the fetus and there are no adequate and well-controlled studies in humans, but potential benefits may warrant use of the drug in pregnant women despite potential risks*), or D (*there is positive evidence of human fetal risk based on adverse reaction data from investigational or marketing experience or studies in humans, but potential benefits may warrant use of the drug in pregnant women despite potential risks*), their use in pregnancy can be contemplated if the potential benefit outweighs the risks. Even more, some of the assayed compounds cross the placenta and, thus, can also benefit the fetus. Nonetheless, if used, this should be done in an individualized way, conditioning dosage and timings, and always under a clinician’s control where the patient is informed of the pros and cons.

Current search for ZIKV antivirals is being conducted with different approaches: by screening of compounds libraries; by the repurposing of drugs of known active efficacy against other diseases now in use in clinical practice, many of which display broad-spectrum activity; and by testing natural products. Two different strategies can be applied when pursuing for antivirals, those searching for compounds directed to viral targets (direct-acting antivirals) and those aimed to target cellular components needed for the viral life cycle (host-directed antivirals).

## 3. Direct-Acting Antivirals

Among the virus-directed drugs tested [18,20] are those acting against the viral RNA-dependent RNA polymerase (non-structural protein 5 (NS5)) catalytic domain, including nucleoside analogs and polymerase inhibitors; the methyltransferase catalytic domain of the NS5 responsible for transferring the mRNA cap; the NS2B-NS3 trypsin-like serine protease needed for proper processing of the viral polyprotein; and the NS3 helicase. The crystal structures of all these proteins have already been resolved and will certainly help to find new antivirals [21,22,23,24,25,26,27,28,29,30,31,32]. In the same way, structures from other viral proteins are also available that could help to design ZIKV therapeutic alternatives, such as those of the capsid C protein [33], whose destabilization may impair ZIKV multiplication, the NS1 [34,35], an immuno-modulator, or the envelope glycoprotein [36,37,38], which mediates cell binding and endosomal fusion, constitutes a major target for neutralizing antibodies, and could be also the target for virucidal compounds [39].

On the other hand, it has also been reported that passive transfer of neutralizing antibodies to pregnant mice suppresses ZIKV multiplication, inhibits cell death, reduces the number of progenitor neuronal cells, and prevents microcephaly [40,41]. Likewise, administration of monoclonal antibodies (MAbs) recognizing the domain III of the ZIKV-E protein protect mice of lethal ZIKV challenge [42,43] and other MAbs are able to bind and neutralize ZIKV, including those directed against the E dimer epitope [44]. Human polyclonal antibodies produced in transchromosomal bovines also protect mice from ZIKV lethal infection, eliminated ZIKV induced tissue damage in the brain and testes, and protected against testicular atrophy [45]. Thus, administration of therapeutic antibodies seems to also be a potential strategy against ZIKV. Nevertheless, it should be noted that, although still controvertial in the case of ZIKV infection [46], the well-known antibody dependent enhancement effect (ADE) [47], of which dengue virus (DENV) is the prototypic model, may potentiate the risk of disease exacerbation.

## 4. Host-Acting Antivirals

Flaviviruses have small RNA genomes (around 10.7 kb in length) and thus require many host factors and co-option of cellular metabolic pathways to successfully infect host cells and propagate efficiently [48]. This offers an opportunity to search for host targets as therapeutic tools that, in many instances, as they are shared by different members of the *Flaviviridae* family, can be envisaged as pan-flaviviral antivirals [48,49,50]. This strategy can be directed to host factors implicated in infection, pathogenesis, and in the immune response, as it has been shown for DENV and the West Nile virus (WNV) [51]. In addition, their effect would be less prone to the emergence of mutants that will escape their action, as often occurs with drugs targeting viral components. Consequently, this kind of approach could ideally lead to the discovery of broad spectrum antivirals that could provide low cost but effective tools for the control of flaviviral threats.

Different approaches are being used to identify potential host factors as therapeutic targets against flaviviruses including the analyses of transcript levels (e.g., next generation RNA sequencing) for altered expression patterns during infection, proteome changes, kinases activities variations, and protein-RNA interactions (e.g., two-hybrid screenings and affinity chromatography). Likewise, functional analysis can be applied by overexpressing cDNAs or by RNAi-mediated loss of function screens using dsRNA, siRNA, or shRNA libraries, although it should be noted that in some cases downregulation is inefficient and some genes have redundant functions [51]. Replicons may also be used to specifically assay replication activity [52,53].

Theoretically, host-acting antivirals can be directed to any molecule or pathway implicated in the different steps of the viral life cycle, from early events (binding, entry, and fusion), to the formation of the replication complex, and the viral maturation and egress.

### 4.1. Early Steps: Binding, Entry, and Endosomal Fusion

**Figure 1:**
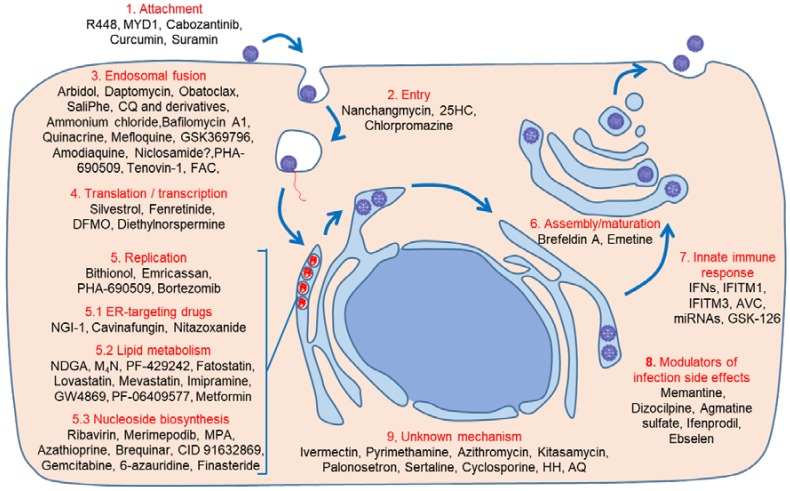
Life cycle of Zika virus (ZIKV) and drugs targeting cellular components. Drugs targeting: attachment (1); entry (2); endosomal fusion (3); translation/transcription (4); replication (5) by affecting the endoplasmic reticulum (ER) (5.1), the lipid metabolism (5.2), the pyrimidine and the purine biosynthesis (5.3); assembly or maturation of the virions (6); or innate immune response (7). Drugs effective for ZIKV infection side effects (8). Drugs with unknown mechanism (9). MYD1: AXL decoy receptor; 25HC: 25-hydroxycholesterol; CQ: chloroquine; FAC: iron salt ferric ammonium citrate; DFMO: difluoromethylornithine; NGI-1: N-linked Glycosylation Inhibitor-1; NDGA: nordihydroguaiaretic acid; M_4_N: terameprocol; MPA: mycophenolic acid; IFNs: interferons; IFITM: interferon-induced transmembrane proteins; AVC: (1-(2-fluorophenyl)-2-(5-isopropyl-1,3,4-thiadiazol-2-yl)-1,2-ihydrochromeno[2,3-c]pyrrole-3,9-dione; HH: hippeastrine hydrobromide; AQ: amodiaquine dihydrochloride dehydrate.

The first step of ZIKV infection is its binding to the cellular receptor (Figure 1). Several molecules have been proposed as a ZIKV receptor (members of the Tyro3/Axl/Mer (TAM) family of receptor tyrosine kinases, T-cell immunoglobulin and mucin domain (TIM) and dendritic cell-specific intercellular adhesion molecule 3-grabbing nonintegrin (DC-SIGN)) that are expressed in different neuronal and non-neuronal permissive cell types. These molecules are also receptors for other viruses, including flaviviruses such as DENV and WNV, regulate several cellular activities (adhesion, migration, proliferation, and survival, release of inflammatory cytokines, antigen uptake, and signaling), and play important roles in the host’s response to infection [54]. However, elimination of a known receptor does not necessarily result in complete protection from viral infection, since flaviviruses use different receptors and, thus, there is always redundancy and alternatives. For instances, inhibiting, downregulating, knocking-down, or ablating AXL, although in some cases they reduce ZIKV infection, they do not completely abolish it, pointing to the use of different cell surface receptors on different cell types [55,56,57,58].

#### 4.1.1. Binding/Entry

Different molecules have been shown to inhibit ZIKV infection at the entry step (Figure 1). R448 (an AXL kinase inhibitor) and MYD1 (an AXL decoy receptor) compromises, but do not completely abolish, ZIKV infection of glial cells [57]. R448, as well as cabozantinib, an inhibitor of AXL phosphorylation, that are currently in clinical trials for anticancer activities, significantly impairs ZIKV infection of human endothelial cells in a dose-dependent manner by affecting a post-binding step [59]. Likewise, curcumin, a widely used food additive and herbal supplement, reduces ZIKV infection in cell culture inhibiting cell binding while maintaining viral RNA integrity [60], as does suramin, an anti-parasitic that interferes with attachment to host cells and with virion biogenesis by affecting glycosylation and maturation [61,62].

#### 4.1.2. Endosomal Fusion

Once ZIKV binds to the cell receptor, like other flaviviruses, it is internalized through clathrin-mediated endocytosis and transported to the endosomes with the involvement of cellular actin and microtubules to establish a productive infection (Figure 1) [57]. After internalization, to start translation and replication, the viral genome is released inside the cytoplasm by fusing the viral envelope with the membranes of the cellular endosomes, a process triggered by acidic pH inside them [63,64]. Nanchangmycin, an insecticide and antibacterial polyether, inhibits ZIKV multiplication and, although the exact mechanism of action has not been completely elucidated, it probably targets AXL and blocks clathrin-mediated endocytosis [65]. Acid endosomal pH triggers rapid conformational changes on viral envelope protein that result in its fusion with endosomal membrane in a pH-dependent manner, thus allowing nucleocapsid release to the cytoplasm for genome uncoating (Figure 1). The optimal pH for conformational rearrangements and viral fusion is 6.3–6.4, and these processes are likely dependent on the presence of cholesterol and specific lipids in the target membrane [66]. These processes can be potentially druggable, and in fact, arbidol, a broad-spectrum antiviral and immunomodulatory use for human influenza A and B infections, inhibits ZIKV multiplication in cell culture probably because it intercalates into membrane lipids leading to the inhibition of membrane fusion between virus particles and plasma membranes, and between virus particles and the membranes of endosomes [67]. Chlorpromazine, an antipsychotic drug that also inhibits clathrin-mediated endocytosis, reduced ZIKV infection, confirming the requirement for clathrin-mediated endocytosis of ZIKV [68]. In addition, 25-hydroxycholesterol (25HC) is increased in ZIKV-infected human embryonic cells and brain organoids, and reduces viremia and viral loads without affecting viral binding, but blocking internalization and suppressing viral and cell membranes fusion [69]. Even more, 25HC reduces mortality and prevents microcephaly in ZIKV-infected mice, and also decreases viral loads in the urine and serum of treated non-human infected primates [69]. Daptomycin, a lipopeptide antibiotic that inserts into cell membranes rich in phosphatidylglycerol, which suggests an effect on late endosomal membranes enriched in this lipid, has also been described as a ZIKV inhibitor [70].

The dependence on endosomal acidification for ZIKV infection also provides a host target suitable for antiviral intervention. For instance, Obatoclax (or GX15-070), an anti-neoplastic and pro-apoptotic inhibitor of the Bcl-2 that targets cellular Mcl-1, impairs ZIKV endocytic uptake by reducing the pH of the endosomal vesicles in cell culture, and thereby most likely inhibits viral fusion [71,72]. However, Obatoclax, which presents a low solubility, has not produced satisfactory results in clinical trials for hematological and myeloid diseases. Saliphenylhalamide (SaliPhe), which targets vacuolar adenosine triphosphatase enzyme (ATPase) and blocks the acidification of endosomes, inhibits ZIKV multiplication in human retinal pigment epithelial cells [71] that are natural targets for ZIKV infection [12]. Similar results were found by Adcock et al. (2017) with SaliPhe using a different screening [73]; however, they reported that, contrary to that described by others [65], other compounds that interfere with the endocytic pathway, such as dynasore, that blocks clathrin-mediated endocytosis, or monensin, a cation transporter, were either toxic for the cells used or did not show any anti-ZIKV activity, as neither did chloroquine (CQ). These contradictory results are probably explained by the different methodologies, cell types, and, to a lower extent, viral strains used to analyze the antiviral activities of the compounds and suggest that compounds showing different activities should be carefully evaluated before going further with investigations. In this line, and contrary to above mentioned report [73], CQ, an FDA-approved anti-inflammatory 4-aminoquinoline and an autophagy inhibitor widely used as an anti-malaria drug that is administered to pregnant women at risk of exposure to Plasmodium parasites, was shown to have anti-ZIKV activity in different cell types (Vero cells, human brain microvascular endothelial cells (hBMECs), and human neural stem cells (NSCs)), affecting early stages of the viral life cycle, possibly by raising the endosomal pH and inhibiting the fusion of the envelope protein to the endosomal membrane [74,75]. CQ has been shown to reduce placental and fetal ZIKV infection [76], and also attenuate ZIKV-associated morbidity and mortality in mice and protect the fetus from microcephaly [77]. Even more, CQ attenuated vertical transmission in ZIKV-infected pregnant interferon signaling-competent Swiss Jim Lambert (SJL) mice, significantly reducing fetal brain viral loads [78]. Similarly, CQ, and other lysosomotropic agents (ammonium chloride, bafilomycin A1, quinacrine, mefloquine, and *N*-tert-Butyl Isoquine (GSK369796)) that neutralize the acidic pH of endosomal compartments, block infection of a human fibroblast cell line and Vero cells [68,75].

Additionally, by medicinal chemistry-driven approaches, a series of new 2,8-bis(trifluoromethyl)quinoline and *N*-(2-(arylmethylimino)ethyl)-7-chloroquinolin-4-amine derivatives have been proved to inhibit ZIKV replication *in vitro* with a higher potency than chloroquine or mefloquine [79,80]. More recently, by screening FDA-approved drugs using a cell-based assay, it has been shown that amodiaquine, another antimalarial drug, also has anti-ZIKV activity in cell culture by targeting early events of the viral replication cycle [81]. Niclosamide, a category B antihelmintic drug approved by FDA, was capable of inhibiting ZIKV infection, and although its antiflaviviral effect has been associated to its ability to neutralize endolysosomal pH and interfere with pH-dependent membrane fusion, in the case of ZIKV, it seems that it was affecting other post-entry steps [82]. In addition, recently, it has been reported that niclosamide decreases ZIKV production, partially restores differentiation, and prevents apoptosis in human induced NSCs; even more, it can partially rescue ZIKV-induced microcephaly and attenuate infection in a developed humanized ZIKV-infected embryo model *in vivo* [83]. Likewise, tenovin-1, which represses cell growth and induces apoptosis in cells expressing p53 by inhibiting the protein-deacetylating activities of SirT1 and SirT2 and, thus, affects endosome functions, potently inhibits ZIKV infection in primary placental fibroblast cells [65]. Iron salt ferric ammonium citrate (FAC) also inhibits ZIKV infection through inducing viral fusion and blocking endosomal viral release by promoting liposome aggregation and intracellular vesicle fusion [84]. Overall, these studies evidence the potential of targeting viral entry to combat ZIKV.

### 4.2. Translation/Transcription

Once ZIKV-RNA is released from the endosomes in the cytoplasm, it acts as mRNA to synthesize the negative-strand viral RNA that directs positive-strand RNA synthesis (Figure 1) [4]. Silvestrol, a natural compound isolated from the plant *Aglaia foveolata* that it is known to inhibit the Asp-Glu-Ala-Asp (DEAD)-box RNA helicase eukaryotic initiation factor-4A (eIF4A) required to unwind structured 5′-untranslated regions and thus impairing RNA translation, exerts a significant inhibition of ZIKV replication in A549 cells and primary human hepatocytes [85]. *N*-(4-hydroxyphenyl) retinamide (fenretinide or 4-HPR), an activator of retinoid receptors that inhibits the proliferation of cancer cells and can induce apoptosis, inhibits ZIKV in cell culture and significantly reduces both serum viremia and brain viral burden in mice by decreasing the rate of viral RNA synthesis, though not via direct inhibition of the activity of the viral replicase [86]. ZIKV relies on polyamines for both translation and transcription [87], so that, drugs targeting the polyamine biosynthetic pathway, such as difluoromethylornithine (DFMO or eflornithine), an FDA-approved drug that is used to treat trypanosomiasis, hirsutism, and some cancers, as well as diethylnorspermine (DENSpm) limit viral replication in BHK-21 cells [88].

### 4.3. Replication, Assembly, and Maturation

ZIKV replication and particle morphogenesis take place associated with a virus-induced organelle-like structure derived from the membrane of the endoplasmic reticulum (ER) (Figure 1) [4]. *De novo* synthesized positive strand-RNA, once packaged, form enveloped immature virions in the ER, enter the secretory pathway and, then, in the trans-Golgi network, the prM is cleaved before the virus is released from the infected cell (Figure 1) [89,90].

ER-membrane multiprotein complexes, such as the oligosaccharyltransferase (OST) complex, have been reported to be critical host factors for flavivirus multiplication. In this regard, it has been shown that the N-linked Glycosylation Inhibitor-1 (NGI-1) chemical modulator of the OST complex blocks ZIKV-RNA replication in different cell types [91]. Similarly, the host ER-associated signal peptidase (SPase) is an essential, membrane-bound serine protease complex involved in cleavage of the signal peptides of newly synthesized secretory and membrane proteins at the ER and also for processing of the flavivirus prM and E structural proteins [92]. It has also been reported that cavinafungin, an alaninal-containing lipopeptide of fungal origin, potently inhibits growth of ZIKV-infected cells [93]. Nitazoxanide, a broad-spectrum antiviral agent approved by the FDA as an antiprotozoan and with potential activity against several viruses in clinical trials (rotavirus and norovirus gastroenteritis, chronic hepatitis B, chronic hepatitis C, and influenza), also inhibits virus infection targeting a post-attachment step, most likely virus genome replication [94]. Likewise, Brefeldin A, a *Penicillium* sp. product that inhibits protein transport from the ER to the Golgi apparatus, inhibits ZIKV multiplication [95], as does Emetine, an anti-protozoal agent that inhibits both ZIKV NS5 polymerase activity and disrupts lysosomal function [96].

ZIKV infection leads to cell-death by inducing host caspase-3 and neuronal apoptosis during its propagation [97]. Thereby, bithionol, a caspase inhibitor, inhibits ZIKV strains of different geographical origin in Vero cells and human astrocytes [98]. Similarly, by using a drug repurposing screening of over 6000 molecules, it was found that emricasan, a pan-caspase inhibitor that restrains ZIKV-induced increases in caspase-3 activity and is currently in phase 2 clinical trials in chronic hepatitis C virus (HCV)-infected patients, protected human cortical neural progenitor cells (NPC) in both monolayer and three-dimensional organoid cultures, showing neuroprotective activity without suppression of viral replication [82]. Additionally, bortezomib, a dipeptide boronate proteasome inhibitor approved for treatment of multiple myeloma and mantle cell non-Hodgkin’s lymphoma that regulates the Bcl-2 family of proteins, has also been described as a ZIKV inhibitor [70]. Similarly, different cyclin-dependent kinase (CDK) inhibitors, such as (alphaS)-4-(Acetylamino)-alpha-methyl-*N*-(5-(1-methylethyl)-2-thiazolyl)benzeneacetamide (PHA-690509), reduced ZIKV-infection and propagation [82]. However, CDK inhibitors should not be suitable for the treatment of pregnant women but could be useful for the treatment of other non-pregnant patients, preventing the complications associated with ZIKV infection.

#### 4.3.1. Lipid Metabolism Modulators

The need for specific host lipids for flavivirus replication and particle envelopment make lipid metabolism a potential target for an antiviral search [66,99], and, even though manipulating a major metabolic pathway such as lipid biosynthesis can be envisaged as a dangerous antiviral approach due to the undesirable effects that could be detrimental for the host, current use of drugs such as ibuprofen and aspirin (cyclooxygenase-2 (COX-2) inhibitors) or statins (3-hidroxi-3-metil-glutaril-CoA (HMG-CoA) reductase inhibitors) highlights the feasibility of lipid-based therapeutics [100,101]. Accordingly, inhibition of key enzymes involved in fatty acid synthesis, such as acetyl-CoA carboxylase (ACC) [102], and fatty acid synthase (FASN) [103,104,105], are potential targets for anti-ZIKV therapy. In this line, we have reported that nordihydroguaiaretic acid (NDGA) and its derivative tetra-O-methyl nordihydroguaiaretic (M4N or terameprocol), two compounds that disturb the lipid metabolism probably by interfering with the sterol regulatory element-binding proteins (SREBP) pathway, inhibit the infection of ZIKV and WNV, likely by impairing viral replication, as did other structurally unrelated inhibitors of the SREBP pathway, such as 4-[(Diethylamino)methyl]-*N*-[2-(2-methoxyphenyl)ethyl]-*N*-(3R)-3-pyrrolidinyl-benzamide dihydrochloride (PF-429242) and fatostatin [106]. In the same way, the dependence on cholesterol for different processes during flavivirus infection also provides a suitable target for antiviral strategies. As mentioned above, 25HC reduces viremia and viral loads *in vitro*, and also reduces mortality and prevent microcephaly in mice, and decreases viral loads in the urine and serum in non-human infected primates [69]. Lovastatin and mevastatin are hypolipidemic agents (HMG-CoA inhibitors) belonging to the family of statins that are widely used for lowering cholesterol in patients with hypercholesterolemia and have been previously shown to present antiviral activity against dengue and hepatitis C viruses. Both agents have been proposed as therapeutic candidates against ZIKV [107]. In fact, lovastatin attenuates nervous injury in animal models of GBS [108]. Likewise, imipramine, an FDA-approved antidepressant, inhibits ZIKV-RNA replication and virion production in human skin fibroblasts, probably by interfering with intracellular cholesterol transport [109]. Regarding sphingolipid metabolism, which has been involved in flavivirus infection [66], treatment with the neutral sphingomyelinase inhibitor GW4869 reduced ZIKV production by affecting viral morphogenesis [110] as described for other flaviviruses [111]. Finally, since adenosine monophosphate-activated protein kinase (AMPK) is a master regulator of lipid metabolism, its activation by PF-06409577 or metformin reduced ZIKV infection by impairing viral replication [112,113]. Thus, targeting lipid metabolism could provide therapeutic alternatives for the discovery of host-directed antivirals against ZIKV.

#### 4.3.2. Nucleosides Biosynthesis Inhibitors

The NS5 protein is the viral RNA-dependent RNA polymerase responsible for the RNA synthesis that also inhibits interferon (IFN) signaling by acting over the signal transducer and activator of transcription 2 (STAT2) protein [114], being, thus, a major target for antiviral design. Besides the proven antiviral activities of different nucleosides analogs and inhibitors of the ZIKV-NS5 [18], several inhibitors of the biosynthesis of nucleosides (purines and pyrimidines) also impair ZIKV replication (Figure 1). Ribavirin is an inhibitor of the inosine monophosphate dehydrogenase (IMPDH) with antiviral activity to several RNA viruses [115], but its mechanism of action is not entirely clear. It may act as a guanosine synthesis inhibitor, a viral cap synthesis inhibitor, a viral RNA mutagen, and as an inducer of lethal mutagenesis [116,117,118]. By using a cell-based assay, no antiviral activity of the drug was initially observed [73] but, later on, it was reported that although no activity against ZIKV was detected in Vero cells, the drug did inhibit virus multiplication in human cell lines, including liver Huh-7 and rhabdomyosarcoma (RD) cells [119]. Further studies have confirmed an inhibitory activity of ribavirin against ZIKV strains of different geographical origin in various types of cells, such as human neural progenitor cells (hNPCs), human dermal fibroblasts (HDFs), human lung adenocarcinoma cells (A549), and even in Vero cells [120,121,122]. Still more, the drug was shown to abrogate viremia in ZIKV-infected STAT-1-deficient mice [121], which lack type I IFN signaling, are highly sensitive to ZIKV infection, and exhibit a lethal outcome. Two other inhibitors of IMPDH, merimepodib (MMPD or VX-497) [123] and mycophenolic acid (MPA) [65,70,124] also inhibit ZIKV-RNA replication in different cell types, including Huh-7 cells, human cervical placental cells, and neural stem and primary amnion cells. However, other authors [73] have described that MPA have little effect on ZIKV replication and showed significant cell toxicity. Likewise, azathioprine, another inhibitor of purine synthesis and immunosuppressant, impaired ZIKV replication in HeLa and JEG3 cells [70]; nonetheless, its use in pregnant women is not recommended. The above described contradictory results stress again the differences that drug treatments may have as a consequence of the different viral strains, cell types, and methodologies used to assess them.

As with the inhibitors of purine biosynthesis, compounds inhibiting the synthesis of pyrimidines have also effect on ZIKV replication (Figure 1). So that, the virus was highly susceptible to brequinar and CID 91632869 treatments in cell culture [73]. However, it should be noted that it has been reported that brequinar, as well as DD264, antiviral activity may not be due to pyrimidine deprivation, but rather to the induction of the cellular immune response [125,126]. Similarly, other inhibitors of the pyrimidine synthesis, such as gemcitabine, an activator of cellular caspases [65,71], and, although with a lower efficiency probably due to its lower solubility, 6-azauridine and finasteride, a 4-azasteroid analog of testosterone that inhibit type II and type III 5α-reductase and is being tested for benign prostatic hyperplasia and male pattern baldness, reduce ZIKV replication [73,107].

#### 4.3.3. Unknown Mechanisms

Several other compounds have been shown to have anti-ZIKV activity by inhibiting viral entry and/or RNA synthesis, although their mechanisms of action have not yet been fully elucidated. Among them are antiparasitics such as ivermectin (used mainly against worms infections) and pyrimethamine (a folic acid antagonist that inhibits the dihydrofolate reductase and, thus, DNA and RNA synthesis, is classified as a pregnancy category C, and was initially used to treat malaria and now toxoplasmosis and cystoisosporiasis) [70]; antibiotics such as azithromycin that prevents infection, replication, and virus-mediated cell dead [55], and kitasamycin (a natural product from Streptomyces narbonensis that inhibits protein biosynthesis) [107]; drugs used to prevent chemotherapy-induced nausea and vomiting as palonosetron (a FDA-approved 5-HT3 antagonist) [107]; antidepressants like sertraline (a selective serotonin reuptake inhibitor) [107] and cyclosporine (that is also use for rheumatoid arthritis, psoriasis, Crohn’s disease, nephrotic syndrome, and in organ transplants, is believed to lower the activity of T-cells, and is currently in clinical trials for tis possible use in ameliorate neuronal cellular damage) [70]. Similarly, after chemical screening, it was found that hippeastrine hydrobromide (HH), an active component of traditional Chinese medicine, and amodiaquine dihydrochloride dihydrate (AQ), an FDA-approved drug for treatment of malaria, inhibit ZIKV infection of human pluripotent stem cell-derived cortical NPCs and in adult mouse brain *in vivo* even when the infection was already ongoing but, again, their mechanisms of action are not known [127].

## 5. Drugs Preventing ZIKV Infection Side Effects

Besides drugs that act against host targets directly implicated in the viral cycle, there are compounds that can prevent undesirable effects of ZIKV infection. In this regard, ZIKV infection leads to massive neuronal damage, especially of neural progenitor cells, and neurodegeneration [128,129,130], via both direct replication in neuronal cells and possibly through increased excitotoxicity via over activation of *N*-methyl-d-aspartate receptor (NMDAR)-dependent neuronal excitotoxicity in nearby cells. Memantine, a pregnancy category B FDA-approved drug widely used to treat patients with Alzheimer’s disease, as well as other NMDAR blockers (dizocilpine, agmatine sulfate, or ifenprodil), prevents neuronal damage and death and intraocular pressure increase induced by ZIKV infection in infected mice, but it does not affect virus replication, pointing to its possible use to prevent or minimize ZIKV-related microcephaly during pregnancy [131]. Ebselen (EBS), an antioxidant that reduces oxidative stress and improves histopathological features in a testicular injury study model and is currently in clinical trials for various diseases, showed minor effects in reducing ZIKV progeny production and viral E protein expression and on overall survival and viremia level of challenged AG129 mice; however, it should be noted that EBS reduced some ZIKV-induced effects, such as testicular oxidative stress, leucocyte infiltration, and production of pro-inflammatory response, whereas, in a model of male-to-female mouse sperm transfer, the drug improved testicular pathology and prevented the sexual transmission of ZIKV [132].

## 6. Innate Immunity Modulation

IFNs play a key role in the elimination of pathogens and they are release upon the activation of the innate immune response by infecting viruses. In this way, ZIKV infection induces IFN signaling pathways and further activates cytoplasmic retinoic acid inducible gene 1 protein (RIG1)-like receptors (RLRs) and several type I and III IFN-stimulated genes, driving to the subsequent activation of the Janus kinase (JAK)/STAT innate immune pathway that confer resistance to ZIKV infection [54]. Different studies showed that IFN-α, IFN-β, and IFN-γ inhibit ZIKV replication in cell culture [124,133,134], and that treatment of pregnant mice with IFN-λ reduced ZIKV infection [135]. In addition, IFITM1 and IFITM3, which are interferon-induced transmembrane proteins, impair early stages of ZIKV infection. Even more, IFITM3 prevents ZIKV-induced cell death [136]. Likewise, it has been reported that an interferon-activating small molecule (1-(2-fluorophenyl)-2-(5-isopropyl-1,3,4-thiadiazol-2-yl)-1,2-ihydrochromeno[2,3-c]pyrrole-3,9-dione (AVC) strongly inhibits replication of ZIKV in cell culture [137]. However, it is also known that the virus is capable of evading type I IFN responses by acting over the JAK/STAT signaling pathway [114,138,139,140], and that type I IFNs might be mediators of pregnancy complications, including spontaneous abortions and growth restriction [141]. In any case, use of IFN against ZIKV, alone or in combination with other antivirals, deserve further studies.

By screening a library of known human microRNAs (miRNAs), small, noncoding RNAs (sncRNAs) that modulate gene expression post-transcriptionally and regulate a broad range of cellular processes, several miRNAs were found to inhibit ZIKV by increasing the capability of infected cells to respond to infection through the interferon-based innate immune pathway [142]. Another alternative is intervening over epigenetic regulation by using epigenetics modulators. For instance, histone H3K27 methyltransferases (EZH1 and EZH2) suppress gene transcription and it has been shown that inhibitors such as 1-[(2S)-butan-2-yl]-*N*-[(4,6-dimethyl-2-oxo-1H-pyridin-3-yl)methyl]-3-methyl-6-(6-piperazin-1-ylpyridin-3-yl)indole-4-carboxamide (GSK-126) reduce ZIKV multiplication in cell culture through the activation of cellular antiviral and immune responses [143]. In any case, further studies are needed to evaluate the potential therapeutic capability of these immunomodulators against ZIKV infection.

## 7. Conclusions

A great effort is being lately made to find compounds to fight ZIKV infection by applying different approaches, from repurposing of drugs with known antiviral activity to the screening of bioactive molecules from different libraries, as well as natural products. However, most of the already tested drugs have been found to inhibit viral replication *in vitro*, and only a few have been tested *in vivo*. Hence, since, in many instances, the results will be difficult to extrapolate to humans, it would be hard for most of the tested antivirals to complete the entire drug development pipeline. In addition, it should be remarked that many drugs could have untoward effects and, thus, careful evaluation should be conducted before using them in clinical practice, as the main target populations for anti-ZIKV therapy will be pregnant women and patients with other medical complications.

Many of the already tested drugs are directed against viral structural and enzymatic proteins, including, for instance, anticancer and anti-inflammatory molecules, antibiotics, and antiparasitics; however, it is well known that this approach can easily lead to the appearance of resistance. Since flaviviruses require many host factors and co-option of cellular metabolic pathways to successfully infect host cells and propagate efficiently, this offers an opportunity to search for host targets as therapeutic tools that, in many instances, can be broad spectrum agents, and which effect would be less prone to the emergence of mutants that will escape their action. Because of that, and even though manipulating host metabolic pathways can be seen as dangerous due to the undesirable effects that could be detrimental for the host, its success for other diseases make of them a realistic option for the treatment of ZIKV infection.
